# Variation in reproductive investment increases body temperature amplitude in a temperate passerine

**DOI:** 10.1007/s00442-021-05026-2

**Published:** 2021-09-07

**Authors:** Jan-Åke Nilsson, Andreas Nord

**Affiliations:** Department of Biology, Section of Evolutionary Ecology, Lund University, Ecology Building, 223 62 Lund, Sweden

**Keywords:** Brood size manipulation, Heterothermy, Hyperthermia, Life history, Thermoregulation, Work rate

## Abstract

Many birds and mammals show substantial circadian variation in body temperature, which has been attributed to fluctuations in ambient temperature and energy reserves. However, to fully understand the variation in body temperature over the course of the day, we also need to consider effects of variation in work rate. We made use of a dataset on body temperature during the resting and active periods in female marsh tits (*Poecile palustris*) that bred in a temperate area and were subjected to experimental changes in reproductive investment through brood size manipulations. Furthermore, the amplitude increased with daytime, but were unaffected by nighttime, ambient temperature. Amplitudes in females with manipulated broods were 44% above predictions based on inter-specific allometric relationships. In extreme cases, amplitudes were > 100% above predicted values. However, no individual female realised the maximum potential amplitude (8.5 °C, i.e. the difference between the highest and lowest body temperature within the population) but seemed to prioritise either a reduction in body temperature at night or an increase in body temperature in the day. This suggests that body temperature amplitude might be constrained by costs that preclude extensive use of both low nighttime and high daytime body temperatures within the same individual. Amplitudes in the range found here (0.5–6.7 °C) have previously mostly been reported from sub-tropical and/or arid habitats. We show that comparable values can also be found amongst birds in relatively cool, temperate regions, partly due to a pronounced increase in body temperature during periods with high work rate.

## Introduction

During recent decades, an increasing number of studies have shown that body temperature of endothermic homeotherms, i.e. birds and mammals, varies much more than previously appreciated (Angiletta et al. 2010). In cold environments, many endotherms reduce body temperature during the night, presumably to save energy (McKechnie and Lovegrove 2002; Ruf and Geiser 2015). In hot environments, endotherms may let body temperature increase above normothermia (Hetem et al. 2010; Nilsson et al. 2016) to save water and energy from reduced cooling demands. Body temperature is also influenced by variation in work rate. Thus, high work rate increases metabolic heat production (Speakman and Król 2010; Nilsson and Nord 2018a) which, if not dissipated, will increase body temperature.

The benefits of a flexible body temperature can easily be interpreted as a means of saving energy. A regulated hypothermic response will offset part of the need for thermogenesis in the cold, and hyperthermia will offset some of the energetically costly and water-consuming heat dissipation processes in warm environments or during hard work. However, the fact that benign ambient temperatures and ample food availability reduce the decrease in nighttime body temperature (Nord et al. 2009, 2011), and that readily available water reduces the extent of hyperthermia (Hetem et al. 2010), suggests that there are physiological costs for deviating from normothermia (Angiletta et al. 2010; Boyles et al. 2011). Thus, endothermic homeotherms may face a trade-off between maintenance of normothermic body temperature and the use of limited energy and water resources for activities other than thermoregulation.

The costs of sustained body temperatures outside normothermia are not well established. In addition to a general reduction in physiological performance (Roti Roti 2008; Boyles et al. 2011), hyperthermia will reduce the oxygen saturation of haemoglobin (Pörtner 2001) and has been suggested to be connected to increased production of reactive oxygen species (Lin et al. 2006; Jimenez and Williams 2014). Torpor, a large reduction of resting body temperature resulting in non-responsiveness to external stimuli (Prinzinger et al. 1991; McKechnie and Lovegrove 2002), has been proposed to cause increased predation risk on account of reduced vigilance (Carr and Lima 2013), at least in species that do not rest in very sheltered locations (cf. Geiser 2020). Even in passerines, an order showing very limited use of torpor (McKechnie and Lovegrove 2002), rest-phase body temperature is higher when perceived predation risk increases (Andreasson et al. 2019) and when the immune system is activated (Nord et al. 2013).

The effect of the thermal environment and activity on circadian variation of body temperature has been measured on an inter-specific scale by estimating the difference between average resting- and active-phase body temperature in non-torpid birds and mammals (Aschoff 1982; Prinzinger et al. 1991). However, very few studies have actually measured body temperature variation in the same individual during the same season and when performing natural behaviours (Lovegrove and Heldmaier 1994; Smit et al. 2013). Population estimates of the potential body temperature amplitude do not necessarily inform us about the amplitude within an individual. To explore the extent of such amplitudes in a natural setting, we used our data on the thermoregulatory consequences of a manipulated increase in parental effort in marsh tits (*Poecile palustris*) (Nilsson and Nord 2017b, 2018b). We measured diurnal and nocturnal cloacal body temperature on the same females and compared the difference between nighttime and daytime body temperature (henceforth body temperature amplitude) to that reported for other groups of birds. Since adult marsh tits with increased parental effort show both a reduction in nighttime and an increase in daytime body temperature (Nilsson and Nord 2017a, 2018a), this dataset provided us with a manipulated increased range of amplitudes. If amplitude within an individual was constrained by ecological or physiological costs, we predict that individual marsh tits would be constrained from using the full range of amplitudes in the population, as measured between individuals. If amplitudes were unconstrained, we predict a negative relation between nighttime and daytime body temperature, reflecting our manipulated variation in parental effort.

## Materials and methods

The analyses in this paper are based on published data of body temperature in female marsh tits breeding in a nest box population in southernmost Sweden where they were subjected to a brood size manipulation experiment (Nilsson and Nord 2017b, 2018b). Marsh tits prefer deciduous woodland and are strictly sedentary (Nilsson 1989) and readily use nest boxes for breeding. Females produce a clutch of 5–11 eggs with annual means ranging from 7 to 9 eggs (Nilsson 1991) which they incubate alone for 12–13 days after which both parents feed the nestlings for 19–21 days (Nilsson and Svensson 1993). During spring, they typically forage by gleaning leaves and branches of shrubs and trees, thus mostly out of direct sunlight.

On day 6 after hatching, we moved 3 or 4 nestlings to create enlarged broods (mean increase: 44.8%; SD = 7.4) and reduced broods (mean decease: 44.3%; SD = 5.5). Un-manipulated broods served as controls. The manipulation resulted in mean brood sizes (± SD) of 4.28 (± 1.25), 7.84 (± 1.48) and 12.41 (± 1.24) in reduced, control and enlarged broods, respectively. See Nilsson and Nord (2017a) for a detailed description of the manipulation.

The data we used here originated from published data of two different body temperature measurement sessions in each female. The first measurement was taken during the night (between 23:10 and 01:50 local time) when females were roosting amongst their 9- or 10-day old nestlings (Nilsson and Nord 2017a). The second measurement was taken during the day when females were feeding their 13- to 19-day-old nestlings (Nilsson and Nord 2018a). Body temperature measurements were obtained using a Testo 925 thermometer (Testo AG, Lenzkirch, Germany) fitted with a 36-gauge type K thermocouple that was inserted 12 mm into the cloaca within 5–10 s of catching the female in the nest box (see Nilsson and Nord 2017a for more details). The specific thermometer and thermocouple combinations were calibrated at 35, 40 and 45 °C by an accredited thermometry laboratory (Nordtec AB, Gothenburg, Sweden) before the start of the experiment. This showed that the instruments were accurate to ± 0.1 °C and we used the corrected values (based on a linear regression between the thermometer reading and the true calibration temperature) in all analyses.

The final sample of females with both day- and nighttime body temperature measurements consisted of 27 observations from enlarged broods (2010: 12; 2011: 15), 34 from control broods (2010: 19; 2011: 15) and 26 from reduced broods (2010: 15; 2011: 11). At night, body temperature averaged 39.87 °C in control females, and it was lower in both those with enlarged (by 0.32 °C) and reduced broods (by 0.41 °C; Nilsson and Nord 2017a). During the day, when feeding nestlings, it averaged 42.92 °C in control females, but was higher in females tending enlarged (by 0.47 °C) and reduced broods (by 0.34 °C; Nilsson and Nord 2018a). From these data, we calculated the difference between diurnal and nocturnal body temperature within a female (henceforth ‘amplitude’). The number of days between the two measurements (mean ± SD: 4.8 ± 0.85; range: 4–10 days; 92% of the observations at 4 or 5 days between measurements) did not affect amplitude (general linear model implemented in SAS PROC GLM: *F*_1,85_ = 0.12; *p* = 0.73). Short-term fluctuations in environmental variables may still have introduced variation in our estimates. The fact that both night- and daytime body temperatures were significantly repeatable on a subsample of females measured the next night or day (*r* = 0.35–072; Nilsson and Nord 2017a, 2018a) indicates that this source of error was probably moderate. The extra disturbance of capturing some females during two consecutive nights did not influence the daytime body temperature measurement (*t* test: *t*_85_ = 1.21; *p* = 0.21). Maximum and minimum daily ambient temperatures were derived from a nearby meteorological station in Lund, 20 km from the study site (Swedish Meteorological and Hydrological Institute, unpublished data).

We analysed data using a linear mixed-effect model fitted using the restricted maximum likelihood method (REML) implemented in SAS PROC MIXED (SAS Institute Inc., Cary, NC, USA). Female identity was used as a random intercept since 12 females bred in both years. We used amplitude as the dependent variable and used year and treatment (enlarged, control and reduced brood) as fixed factors. Minimum ambient temperature during the night of measurement (mean ± SD: 9.37 ± 2.46; range 2.7–13.6 °C) and maximum daytime ambient temperature (mean ± SD: 18.57 ± 2.66; range 14.2–26.1 °C) at the day of measurement were included as covariates. The two measurements of ambient temperature were not significantly correlated (general linear model: *F*_1,87_ = 2.76; *p* = 0.10). In addition, we added all interactions between treatment and the other explanatory factors. To further explore how individual variation in night- and daytime body temperatures affected amplitude, we performed a mixed-effect model (SAS PROC MIXED) with daytime body temperature as the dependent variable and nighttime body temperature, treatment and nighttime body temperature × treatment as explanatory variables. Female identity was used as a random intercept. Degrees of freedom for all mixed models were calculated using the Satterthwaite approximation. Post hoc comparisons were performed using the Tukey HSD method.

## Results

**Fig. 1:**
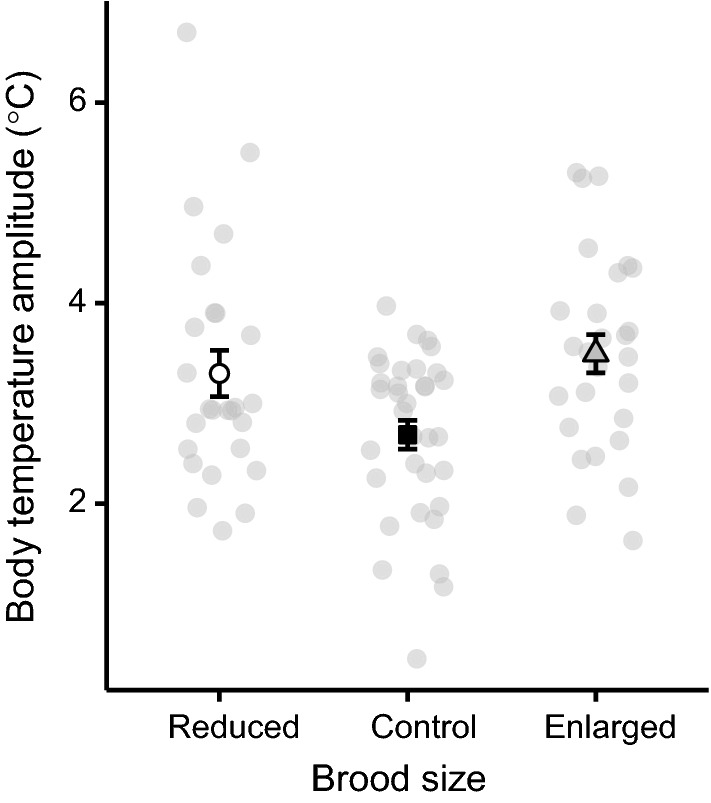
Mean ± SE difference in body temperature between days and nights (i.e. the body temperature amplitude) in female marsh tits in relation to experimental brood size category (reduced, control, enlarged). The grey points behind the error bars show raw data

**Fig. 2:**
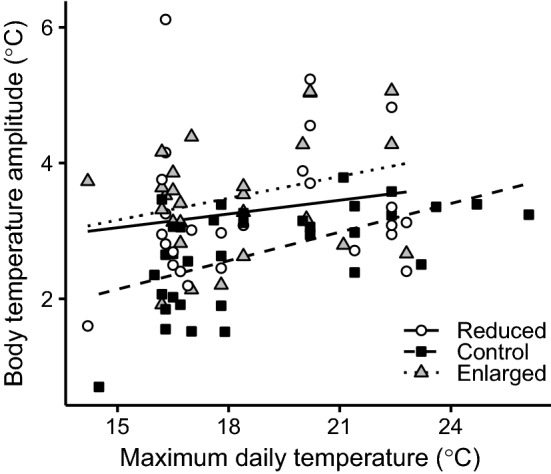
The predicted difference in body temperature between days and nights (i.e. the body temperature amplitude) in female marsh tits in relation to maximum daily temperature

The mean amplitude of females tending un-manipulated control broods was 2.69 °C (SD = 0.83; range 0.45–3.97 °C). However, the amplitude of females tending both enlarged (least squares mean = 3.53 °C; SE = 0.16; post hoc test: *p* = 0.0003) and reduced (least squares mean = 3.41 °C; SE = 0.17; post hoc test: *p* = 0.0022) broods were significantly larger than females caring for control (least squares mean = 2.75 °C; SE = 0.15) broods (Table 1; Fig. 1). There was no difference in amplitude between the two brood-manipulated categories (post hoc test: *p* = 0.58). Five females had body temperature amplitudes surpassing 5 °C, with one female reaching 6.7 °C (Fig. 1). The body temperature amplitude increased with increasing maximum daytime ambient temperature (Table 1; Fig. 2) but nighttime minimum temperature did not affect the amplitude (Table 1). Amplitudes were also smaller on average in 2010 than in 2011 (Table 1). None of the interactions between treatment and the other explanatory factors was significant (all *p* > 0.29) and is not included in the final model (Table 1).

**Table 1:** Tests of the effect of maximum day temperature, minimum night temperature, year (2010 and 2011) and treatment (females tended enlarged, control or reduced broods) on the difference in body temperature between days and nights (‘body temperature amplitude) in female marsh tits

Dependent variable	Explanatory variable	Estimate (± SE)	*F*	d.f.	*p*
*Body temperature amplitude*	Maximum daily temp	0.211 (0.033)	13.04	1,50.5	**0.0007**
	Minimum nightly temp	0.065 (0.042)	2.35	1,60.6	0.13
	Year		8.30	1,48	**0.0059**
	2010	2.97 (0.14)			
	2011	3.49 (0.14)			
	Treatment		9.84	2,37.5	**0.0004**
	Enlarged	3.53 (0.16)			
	Control	2.75 (0.15)			
	Reduced	3.41 (0.17)			

Estimates (± SE), test statistics, degrees of freedom and *p* values are shown, with significant *p* values marked in bold font

**Fig. 3:**
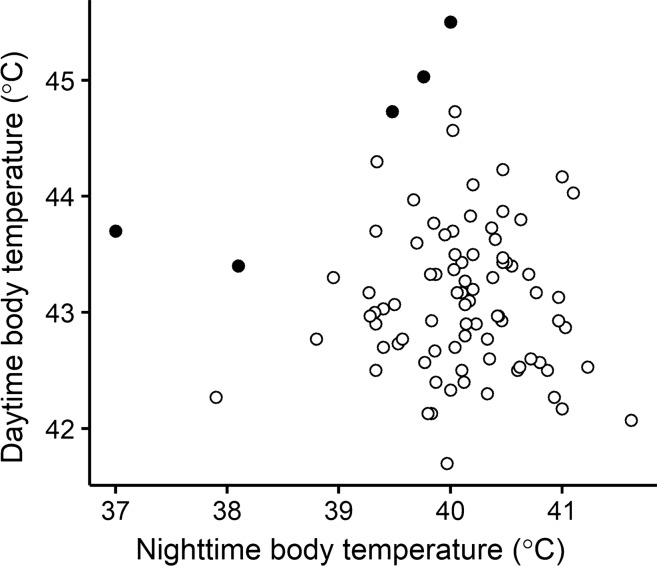
The relationship between daytime and nighttime body temperatures (°C) in female marsh tits. The black triangles show the five individuals where amplitude was higher than 5 °C

The relationship between daytime and nighttime body temperature did not differ between the experimental treatments (i.e. nighttime body temperature × treatment: *F*_2,79.9_ = 0.05; *p* = 0.95). Nor did the main effect of nighttime body temperature significantly affect daytime body temperature (*F*_1,84.5_ = 0.65; slope = − 0.086 (SE = 0.107); *p* = 0.42; Fig. 3). As expected, treatment affected daytime body temperature (*F*_2,72.4_ = 3.79; *p* = 0.027), irrespective of nighttime body temperature.

## Discussion

We found that the circadian amplitude of body temperature increased substantially in individual marsh tit females when tending manipulated broods. The high demand for parental effort when feeding enlarged broods prompts a reduction in nighttime body temperature to compensate for a shift in the trade-off from self- to increased nestling feeding during the day (Nilsson and Nord 2017a). On the other hand, the high feeding rate that is necessary to cover nestling demand in enlarged broods will induce higher daytime body temperatures due to increased metabolic heat production and insufficient heat dissipation rate when flying (Nilsson and Nord 2018a; Nord and Nilsson 2019). The large amplitude in females tending reduced broods, which is indicative of increased energy expenditure despite lower feeding rate, has been hypothesised to depend on the onset of other energetically costly processes. One such activity is breeding-moult overlap, which has been experimentally shown to coincide with relieved feeding duties (Svensson and Nilsson 1997) and result in enhanced future survival (Morales et al. 2007). Increased amplitude in the reduced females could also be related to the brood size manipulation per se. Although the disturbance in connection to the manipulation was the same in all experimental categories (Nilsson and Nord 2017a), females could have perceived the reduction in brood size as a predation attempt (Nilsson and Nord 2018a). Increased predation risk has been shown to increase chronic stress levels (Clinchy et al. 2004), which has been suggested to be related to increased energy expenditure (Jimeno et al. 2017). The impact of experimental brood reduction on the physiological processes of parents needs to be addressed in further studies.

The amplitude of control females (2.69 °C) during normal activity was comparable to allometric predictions for marsh tit-sized birds (mean mass = 11.20 g; SD = 0.38; range 10.2–12.0 g for females in this study) based on both Ashoff (1982) (3.1 °C, *n* = 21 species) and Prinzinger et al. (1991) (2.43 °C, *n* = 202 species). Compared to the more exhaustive study by Prinzinger et al. (1991), females tending manipulated broods had body temperature amplitudes that were, on average, 44% higher than predicted values. The five most extreme females had amplitudes > 106% above predictions (Prinzinger et al. 1991). These high amplitudes are due to both a reduction in nighttime and an increase in daytime body temperature (Nilsson and Nord 2017a, 2018a).

By manipulating brood size and thereby increase energy demand, we assumed that individuals with the highest daytime body temperatures (Nilsson and Nord 2018a) also would be those with the lowest nighttime body temperatures (Nilsson and Nord 2017a) since they needed to conserve energy the most. Following from this, we would predict a negative relation between these two body temperatures within a female. However, this would only be true if there are constraints on the amplitude that can be accommodated within an individual. Our finding that the relationship between the two body temperatures was far from significant (Fig. 3) indicates that physiological or ecological costs constrain amplitudes within individuals. Thus, the females with the highest body temperature during the day were not necessarily those with the lowest nighttime body temperatures. The five females with amplitudes > 5 °C illustrate this point: two of them showed low nighttime body temperatures but moderate daytime temperatures, whereas the three with high daytime body temperatures showed moderate nighttime temperatures (Fig. 3). It is tempting to speculate that the cost of amplitudes does not increase linearly, such that engaging in both low nighttime and high daytime body temperatures within a short time period is too costly. In the case of the marsh tit, this amplitude may be in the range of 5 °C. Although the between-female amplitude in the population was 8.5 °C (Fig. 3), this was far from realised *within* any individual. Further studies should address which strategic or state factors that explain whether females prioritise low body temperature to save energy at night or high body temperature to allow a high sustained work load during the day.

Few studies have measured body temperature in the same individuals over the circadian cycle in birds under natural conditions. In passerines, captive starlings (*Sturnus vulgaris*) under normal photoperiod in a temperate region had amplitudes ranging between 2.6 and 3.0 °C, which increased to 3.6 °C under a winter-like photoperiod (Dawson 2017). Free-living white-browed sparrow-weavers (*Plocepasser mahali*) in the Kalahari Desert, South Africa, had a body temperature amplitude of 4.5 °C (Smit et al. 2013). Studies on non-passerines in sub-tropical environments also report body temperature amplitudes of between 4.2 and 5.5 °C (McKechnie and Smit 2010; Kemp et al. 2017). Thus, high body temperature amplitudes have mostly been described for birds living in sub-tropical and/or in arid environments. Our study indicates that such high body temperature amplitudes can also be the result of high-energy expenditure, even when birds inhabit cool temperate regions. Since these estimates were based on a single body temperature measurement during night and day, the true maximum amplitude may have been even higher.

Low body temperatures during the night contributed more to body temperature amplitude than did high daytime body temperatures in the desert-dwelling white-browed sparrow-weaver (Smit et al. 2013). In the present study, low nighttime body temperature added more to amplitude in some individuals whereas high daytime body temperatures contributed more in others. However, since amplitude increased with diurnal, but not with nocturnal, ambient temperature, conditions during the day probably influenced the amplitude the most. In arid regions, where temperature often varies greatly between night and day, it has been suggested that cold nights are sometimes harder to deal with than warm days, at least if drinking water is available (Cooper et al. 2019). This might apply particularly to non-breeding birds, such as the white-browed sparrow-weavers in the study by Smit et al. (2013), because they are able to restrict foraging to shaded areas and avoid excessive activity during the hottest parts of the day. A similar mechanism may explain the observation of a higher body temperature, and resultant higher body temperature amplitude, in subordinate compared to dominant birds in other studies (Cunningham et al. 2017). Breeding marsh tits, on the other hand, have less opportunities to restrict foraging effort without paying a disproportionate cost in terms of reduced nestling condition. Furthermore, behaviours promoting evaporative heat loss, such as panting and wing spreading, are probably hard to combine with intensive foraging (Du Plessis et al. 2012, van de Ven et al. 2019). Besides the need to forage also in hot weather, breeding birds produce considerable amount of excess heat when flying back and forth to the nest feeding nestlings. Failure to dissipate this heat will increase body temperature to a point where foraging rate might be compromised (Speakman and Król 2010; Andreasson et al. 2020a). In line with this, experiments designed to facilitate heat dissipation have resulted in changes to the trade-off between reproduction and self-maintenance. Facilitation of heat dissipation either increased aspects of self-maintenance (Nord and Nilsson 2019; Andreasson et al. 2020b) or increased nestling feeding rate (Tapper et al. 2020) and nestling quality (Nord and Nilsson 2019).

We conclude that the amplitude in body temperature is potentially driven by environmental factors that sometimes favours energy conservation during the night, sometimes leads to a work-induced increase in body temperature, and sometimes a combination of the two. This is corroborated by fasting experiments, where decreases in nighttime body temperature adds much more to body temperature amplitudes than do changes in daytime body temperature (e.g. Noakes et al. 2013). In line with this, white-browed sparrow-weavers increased their amplitude during dry periods with a probable reduction of food and water resources (Smit et al. 2013) and marsh tits manipulated to increase their parental effort increased their body temperature amplitude (Nilsson and Nord 2018a). Future studies should investigate the constraints imposed on this trade-off, including the proximate cost functions that give preference for low nighttime or high daytime body temperatures in different environmental conditions and in relation to life history trade-offs.

## Data Availability

The datasets supporting this this article are available at Dryad Digital Repository; https://doi.org/10.5061/dryad.4k0j7 (Nilsson and Nord 2017b) and https://doi.org/10.5061/dryad.2q2h5 (Nilsson and Nord 2018b).
